# The role of TREM2 in sepsis

**DOI:** 10.1172/JCI188895

**Published:** 2025-02-17

**Authors:** Jiejie Zhu, Tianyin Sun, Hanren Dai

**Affiliations:** 1 Department of Gastroenterology, The First Affiliated Hospital of Anhui Medical University, Hefei City, Anhui Province, China.; 2 School of Pharmacy, Inflammation and Immune-Mediated Diseases Laboratory of Anhui Province, Anhui Medical University, Hefei, China.

**Keywords:** Infectious disease, Therapeutics, Macrophages

**To the editor:** Sepsis is a major health problem that is characterized by a documented infection and systemic inflammatory response (1). We read with great interest the recent article by Ming et al. (2), wherein the authors revealed the role of TREM2 in aggravating sepsis by inhibiting fatty acid oxidation, which may provide a therapeutic target for sepsis manipulation. In this study, Ming et al. used wild-type (WT) and TREM2-knockout (TREM2^–/–^) mice to establish mouse models of sepsis induced by cecal ligation and puncture (CLP). The authors found that TREM2 knockout improved the survival rates and reduced the levels of inflammation and organ injuries in the CLP model.

However, we have some concerns about their data and conclusions. We found that their findings were contradictory to the study by Zhang et al. published in *Nature Metabolism* (3). Zhang et al. also employed WT and TREM2^−/−^ mice to establish a CLP-induced mouse model of sepsis. The TREM2^−/−^ mice used in these two studies were both provided by Prof. Marco Colonna (Washington University, St. Louis, Missouri, USA). Zhang et al. analyzed the survival of WT (*n* = 18) and TREM2^−/−^ (*n* = 30) mice after CLP, and found that the mortality of TREM2^−/−^ septic mice was notably increased and the levels of proinflammatory mediators were higher after CLP. However, we could not ascertain the number of surviving mice reported by Ming et al., and all the WT mice were apparently dead 72 hours after CLP challenge. Moreover, experiments detecting lung injuries and inflammatory cell infiltration were focused on a limited number (*n* = 5) of animals in each group, while the research by Zhang et al. was based on a larger number of mice.

## Version 1 02/17/2025

Electronic publication
